# Connecting the pieces: Using ORCIDs to improve research impact and repositories

**DOI:** 10.12688/f1000research.6502.1

**Published:** 2015-07-07

**Authors:** Mohamed Baessa, Thibaut Lery, Daryl Grenz, J. K. Vijayakumar

**Affiliations:** 1 King Abdullah University of Science and Technology (KAUST), KAUST Library, Thuwal, 23955-6900, Saudi Arabia; 2 King Abdullah University of Science and Technology (KAUST), Office of Sponsored Research, Thuwal, 23955-6900, Saudi Arabia

**Keywords:** ORCID, Research Impact, Research repositories, KAUST, CRIS

## Abstract

Quantitative data are crucial in the assessment of research impact in the academic world. However, as a young university created in 2009, King Abdullah University of Science and Technology (KAUST) needs to aggregate bibliometrics from researchers coming from diverse origins, not necessarily with the proper affiliations. In this context, the University has launched an institutional repository in September 2012 with the objectives of creating a home for the intellectual outputs of KAUST researchers. Later, the university adopted the first mandated institutional open access policy in the Arab region, effective June 31, 2014. Several projects were then initiated in order to accurately identify the research being done by KAUST authors and bring it into the repository in accordance with the open access policy.

Integration with ORCID has been a key element in this process and the best way to ensure data quality for researcher’s scientific contributions. It included the systematic inclusion and creation, if necessary, of ORCID identifiers in the existing repository system, an institutional membership in ORCID, and the creation of dedicated integration tools. In addition and in cooperation with the Office of Research Evaluation, the Library worked at implementing a Current Research Information System (CRIS) as a standardized common resource to monitor KAUST research outputs. We will present our findings about the CRIS implementation, the ORCID API, the repository statistics as well as our approach in conducting the assessment of research impact in terms of usage by the global research community.

## Introduction

The quality of a research institute can be defined in many ways; quality of its teaching, graduates, research outputs, social, environmental and economic impacts. Indeed, many of these attributes are incorporated into various international ranking formulae which are beginning to garner more and more media and political attention year on year. In this context, it is important for a young university, such as King Abdullah University of Science and Technology (KAUST), created 5 years ago, to gather, curate, monitor and analyze all the publications produced by its researchers. Recognizing the fundamental importance of preserving and sharing such knowledge, the University Library launched its digital repository services in September 2012 (available at http://repository.kaust.edu.sa/kaust/). As correctly mentioned by Richard K. Johnson (The former library reference and collection manager 2009 – 2013) “University rankings typically look at the volume and influence of an institution’s research output, when works are openly accessible on the Internet, they get more citations. And citations are the whole game”. We present here our approach to the objectives and ensuing policies of an academic repository, its integration with other academic systems, its usage as a research evaluation tool and its importance for the university.

The repository ( http://repository.kaust.edu.sa/kaust/) services aim to bring together all of the university’s intellectual output, published and unpublished works alike. After several months of evaluation and feasibility study, the library and IT teams evaluated a number of issues (e.g., technology selection, hosting strategy, depositor authentication) and recruited and trained staff for the program. The following three objectives were established for the repository [ http://www.dpconline.org/newsroom/whats-new/933-whats-new-issue-50-nov-2012#oneworld]:

➢ Provide persistent access to university intellectual assets, including grey literature (e.g., technical reports, conference papers, theses etc.) and research data, in order to preserve and share scientific knowledge created at KAUST.
➢ Showcase the intellectual output of KAUST research, the development of international research networks and collaborations, and support graduate student and post-doc recruitment.
➢ Expand the impact of KAUST research, which contributes to increased awareness of and growing prestige for our new and ambitious university.

The real challenge for any repository initiative lies in trying to serve the needs of a variety of stakeholders. Dealing with this challenge requires identifying different groups of stakeholders, understanding their motivations and needs, and then striving to meet their aspirations. For example, researchers’ concerns lie with the impact of their research within the scholarly community; in order to have an impact, their research outputs in the repository need to be discoverable in ways that allow them to be disseminated widely among the relevant community of researchers. [ http://www.sparc.arl.org/sites/default/files/ir_final_release_102.pdf]

On the other hand, the research evaluation office seeks to locate an accurate and comprehensive list of different university research outputs, to be able to analyze it and better evaluate the effectiveness and significance of research activities.

The library has taken different approaches to building the collection and to meeting the needs and concerns of varied stakeholders. This paper will discuss policy and technology developments and their role in building the repository collections, how ORCID implementation is helping in locating and linking research, how these efforts relate to research evaluation at KAUST, and finally will discuss the role of the library in engaging the community to learn about related issues.

## Development of repository policies

Policy plays an important role in terms of the repository collections development by granting authority and shaping the framework of assigned roles and responsibilities. The primary relevant policies so far have been for management of theses and dissertations, and for open access to published research.

## Electronic theses and dissertations policy

**Figure 1.**
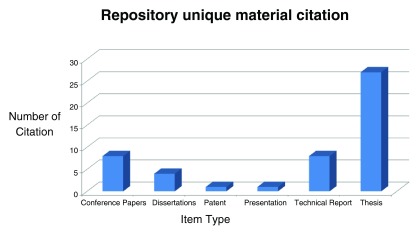
Citation of unique repository materials, according to Google Scholar.

Theses and dissertations represent the scholarly achievement of KAUST students, and from the beginning the university has made a decision to capture, preserve, and provide access only to the electronic versions of this work, with no printed versions required. Theses and dissertations were managed within SharePoint (Microsoft) for the first two years while decisions were made regarding a long-term policy and platform. With the decision in 2012 to move theses and dissertations to the repository and provide public access, the theses and dissertations policy addressed issues like copyright while also setting clear roles and responsibilities [ https://www2.le.ac.uk/library/downloads/etd2014/etds-in-a-new-university], the following figure, Figure 1 demonstrates some unique repository materials that have been cited.

The electronic theses and dissertations policy retains a non-exclusive license for the university to make dissertations or theses publicly available, with an option for the student to embargo public access for one year. The submission workflow has also been standardized, with students submitting the final version of their work to their graduate program coordinators, who then deposit the files in the repository, where the deposit is checked by the repository team before being made publicly available. The repository now has 338 theses and 80 dissertations made available through this policy, with approximately 30 citations.

Unique repository materials citations Source: Google Scholar, Tools: publish or perish http://www.harzing.com/pop_win.htm, Coverage: until May 3, 2005 [ Baessa *et al*., 2015]. Click here for additional data file. Copyright: © 2015 Baessa M et al. 2015 Data associated with the article are available under the terms of the Creative Commons Zero "No rights reserved" data waiver (CC0 1.0 Public domain dedication).

## Open access policy

The rise of the concept of open access to research or scholarly output has led many funding bodies, institutions, and governments to adopt open access policies [ http://legacy.earlham.edu/~peters/fos/newsletter/02-02-09.htm#choicepoints]. When KAUST adopted an open access policy covering published research in June 2014, it became the first proponent of such a policy in the Middle East [ http://hdl.handle.net/10754/337608].

**Figure 2.**
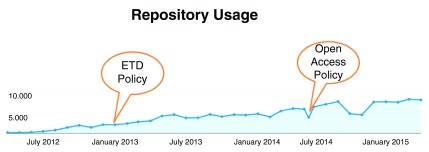
Repository usage from 2012 to the present. KAUST repository usage captured from Google Analytics.

Under the policy each KAUST author grants a non-exclusive license to the university to exercise all rights under copyright relating to each of the authors’ scholarly research articles for the purpose of open dissemination. A committee consisting of faculty, library and legal office representatives, conducted over 15 meetings to address all issues and concerns prior to adoption of the policy. The result was a carefully balanced policy and supporting procedures and tools, including an author addendum to notify a publisher of the policy’s existence, an automated waiver form for a researcher to waive the open access (but not the deposit) requirement of the policy, and a cover sheet on repository items to support version awareness and accuracy in citation. The repository now has full-text deposits of over 1100 research publications, approximately 400 of which are items published after adoption of the open access policy. As illustrated on Antelman case study Open access publishing may reach more readers, the following Figure 2 shows the effect of policy implementation on usage of the repository content.

##  Repository integration with external systems

The adoption of the KAUST open access policy was just part of a broader expansion of the KAUST research repository towards being a reliably comprehensive resource for all of the university’s research outputs. This expansion has necessitated efforts to track the appearance of KAUST research in external systems and to bring information from external systems into the repository in order to properly contextualize the archived research.

## Use of ORCID IDs

One significant effort has been the integration of ORCID IDs into the KAUST repository. This began in August 2014 with the addition of features to our DSpace repository software allowing for the entry and display of ORCID IDs. This development work was undertaken by our hosting provider Biomed Central and consisted of the following elements:

- Addition of ORCID IDs along with the names of various contributor types (authors, thesis/dissertation advisors, and thesis/dissertation committee members) either through the item submission form or through the metadata editing functions.
- Options for retrieval of ORCID IDs during item submission, either via Crossref (if ORCID IDs are available in the metadata associated with a DOI) or via the ORCID public API (based on name search and selection).
- Display of ORCID IDs on the individual item pages and in the browse authors listing along with links to search the repository by the ORCID ID and to the matching ORCID profile.

At this time we also instituted a policy of requiring ORCID IDs to be added for the student authors of newly deposited theses and dissertations in the repository. User expectations that use of ORCID IDs would have additional benefits (such as the automatic addition of thesis information to ORCID profile pages) contributed to our decision at this time to pursue ORCID membership. Our goals for institutional membership in ORCID included being able to assist all KAUST-affiliated authors with the registration of ORCID IDs and also help them derive benefits from the use of ORCID by making it easier to maintain up-to-date publication lists in their public profiles by pushing work information from the KAUST repository into their ORCID profiles.

We then explored the relative benefits of creating and hosting our own integration with the ORCID member API or pursuing integration through our repository provider ( Biomed Central’s Open Repository) or through our CRIS provider ( Elsevier’s Pure). After investigation we decided that developing our own integration would allow us to more rapidly make effective use of our ORCID membership while preserving our options for moving to as-yet-to-be-developed integrations from commercial providers at a later date.

## Use of the ORCID member API

Our internally developed integration with the ORCID member API provides a web interface built with PHP on a MySQL backend through which users can interact with the ORCID system to create an ORCID ID and grant permissions to KAUST to add work and affiliation information to their ORCID profiles. We launched this service in January 2015 with emails to faculty explaining the benefits of ORCID and providing a custom link to our intranet tool for creation of a new ORCID ID or connection of an existing ORCID ID. We then followed up with additional email communication to those who did not use the tool in response to the first email. We have since followed the same process with postdoctoral researchers and research scientists at KAUST. Our current uptake rates are 82% for faculty, 52% for postdocs and 13% for research scientists (who were first contacted at the end of March). Our plan is to implement a similar process for current students while also working internally to include ORCID creation in the procedures for incoming faculty, staff and students in the future.

The functionality of adding work information from the KAUST repository to ORCID profiles by using the KAUST/ORCID integration tool has been well received and has prompted some individuals to deposit works in the repository. However, users have also indicated a desire for additional functionality, allowing them to easily reuse their ORCID work lists on their personal web pages or in other online profiling systems.

## Inclusion of sources of research usage metrics

As we explore ways to derive benefits from ORCID use, we have been looking specifically at how external systems can be leveraged to answer questions about how, and how much, KAUST research outputs are being used. The repository statistics themselves are derived from Google Analytics and provide indicators of the most used items within the repository. On our request our repository provider has also added Scopus citation counts and the Altmetric.com donut badge to the display page for individual items in the repository. In order to have a more comprehensive view of research usage we are also procuring a subscription to Ebsco’s PlumX service.

We made the decision to use PlumX after evaluation of it in comparison with a similar product from Altmetric.com called Altmetric for Institutions. While there is some overlap in the metrics types and sources used by the two services (primarily those from social media sites), we also found significant differences. Altmetric.com has emphasized finding meaningful mentions of published articles and conference papers in the news media, on blogs, and in government documents. However, the broader net that PlumX throws in other areas struck us as likely to give us a more comprehensive view of the varied types of research output produced by KAUST authors (such as computer code, datasets, videos and presentations), and also the varied metrics associated with their usage (downloads, views, citations, etc.). PlumX’s use of the ORCID public API to remain regularly updated with new works added to a researcher’s profile also presents an attractive method of showing researchers a benefit of using the KAUST repository, ORCID and the KAUST/ORCID integration.

## Moving towards use of a CRIS system

A key feature of our future university research tracking and evaluation system will be a current research information system that may become the central locus for information exchange between these varied systems (the repository, ORCID, PlumX, etc.). The Research Evaluation Office has taken the lead in this area by purchasing the Pure system from Elsevier. The initial work has focused on integrating with KAUST’s internal information systems, after completion of which the groundwork will be laid for university-wide use and implementation of connections to external systems to better assess the quality of research done by our faculty. Indeed, we have more than 130 professors coming from all around the world, bringing with them their personal academic and bibliographical history. As a consequence, accurately collecting all the present and past information is a real challenge. Additionally, most of our researchers are involved in collaborations with top scientists worldwide. This brings another degree of complexity when dealing with the measurement of outcomes and their impact.

The integration of ORCID in our system will help us to reduce the errors of affiliations of researchers by giving them a unique and centrally managed identifier. We are starting to use the ORCID identifier connected to our internal reference as the entry point for our users in the Pure system. To evaluate researcher publication records we collect a full list of publications from the researchers themselves and check their validity in Web of Science, Scopus and other online databases. We include the publications prior to their start at KAUST in order to assess the relative productivity of researchers since their arrival compared to their previous career. Unfortunately, the common online tools such as Scopus, Scival, Web of Science or InCites do not have the flexibility to refine the analysis to include start and end dates of affiliations and other research outputs, such as grey literature.

As an example, we present here an analysis of the number of citations per publication for institutional, national, international and corporate collaborative outputs for 86 Institutions that had significant collaborations (generating 10 publications or more) in place with KAUST researchers over the period of 2011–2013. The sample set of institutions was diverse in both geographical location and international ranking and as such provided a representative world average.

**Figure 3.**
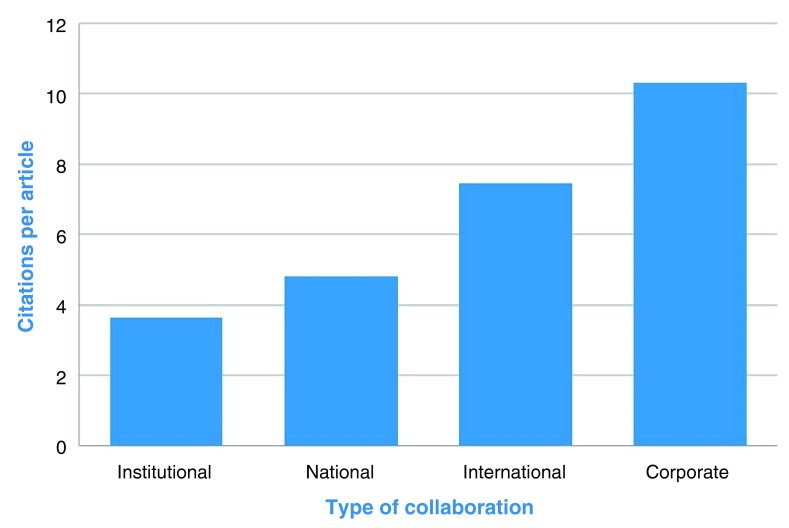
Average number of citations per article for different types of collaboration for the top institutions working with KAUST, as given by Scopus between 2011 and 2013.

On average the quality/impact of a publication (as measured in citations per article) was higher when the publication included a national (4.8) or international (7.4) collaborator ( Figure 3). Of particular note, those publications that included a corporate collaborator (10.3) were on average the most impactful, having the highest average citations per publication, while institutional collaborations (3.6) had the lowest number of citations. These results demonstrate that international scientific collaborations with KAUST can be seen as drivers to international impact and prestige, and that such an impact increases with the number of partners and the industry involvement within those collaborations.

**Figure 4.**
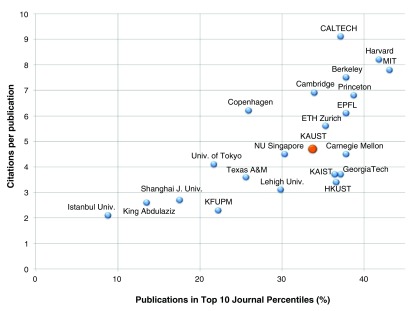
Percentage of publications in top 10 percentile journals with respect to citations per publication for various universities including KAUST.

In terms of research assessment, the main key indicators that we are using are the Field Weighted Citation Impact, the average number of citations per publication, the percentage of publications in top percentile journals and the number of collaborative publications. In this context, we have compiled results for several universities to benchmark KAUST against other similar institutions, as presented in Figure 4.

Bibliometric benchmarking of KAUST University compared to other similar institutions Abbreviations:
CALTECH; California Institute of Technology
Carnegie Mellon; Carnegie Mellon University
EPFL; Ecole polytechnique federale de Lausanne
ETH Zurich; Eidgenessische Technische Hochschule Zurich
GeorgiaTech; Georgia Institute of Technology
Harvard; Harvard University
HKUST; Hong Kong University of Science and Technology
Istanbul Univ.; Istanbul University
King Abdulaziz; King Abdulaziz University
KAUST; King Abdullah University of Science and Technology
KFUPM; King Fahd University of Petroleum and Minerals
KAIST; Korea Advanced Institute of Science and Technology
Lehigh Univ.; Lehigh University
MIT; Massachusetts Institute of Technology
NU Singapore; National University of Singapore
Princeton; Princeton University
Shanghai J. Univ.; Shanghai Jiao Tong University
Texas A&M; Texas A&M University
Berkeley; University of Berkeley
Cambridge; University of Cambridge
Copenhagen; University of Copenhagen
Univ. of Tokyo; University of Tokyo [ Baessa *et al*., 2015] Click here for additional data file. Copyright: © 2015 Baessa M et al. 2015 Data associated with the article are available under the terms of the Creative Commons Zero "No rights reserved" data waiver (CC0 1.0 Public domain dedication).

Thanks to the library repository, the central CRIS system and our internal analysts, those results have allowed the senior management to understand the quality of the research performed at KAUST as well as its impact at the international level. As a consequence, our researchers are also taking a more active part in the collection of data about their publications and other research outputs.

## Community engagement: role of the university library

The roles of the university library are changing in relation to scholarly communications and academic research practices [ Malenfant, 2010]. At KAUST University Library we are actively exploring new roles that we can play in relation to storage and dissemination of all forms of scholarly research with an overall goal of increasing the impact that KAUST research has in the global research community. We also support university stakeholders as they gather information about the impact of KAUST research and select tools to evaluate this information. A key element of this work is interacting with students, researchers and faculty to introduce the available tools for measuring and monitoring the impact of research, while also answering questions about how, when and why to use a given tool.

Along with helping individuals with queries the library has also developed a program of scheduled workshops to be held every semester. Below is a summary of the relevant areas covered in these sessions:

- Value of using citation databases in the literature search
- Understanding citation metrics and tools (h-Index, Impact Factor, Altmetrics etc.)
- Role of publications in effecting institutional rankings
- Understanding researcher profiling (ORCID, Google Scholar etc.)
- Benefits of open access and explanation of how to use the institutional repository
- Academic honesty, plagiarism, and the use of similarity checking tools

Feedback received from participants shows that they found the trainings beneficial to their understanding of how to communicate their research. Based on the positive feedback received, the library is planning to offer additional training to support researchers in navigating confusing and complex aspects of the changing scholarly communications landscape while connecting the pieces of the research life cycle, and thus enhancing the profile of individual researchers, increasing the impact of their research and improving the reputation of our institution.

The KAUST Library also conducts regional outreach in the Middle East to promote open access and research service initiatives. We shared our experiences at the Special Library Association Arab Gulf Chapter conferences in two panels (2013 and 2014) and at the American Library Association’s Sharjah Book Fair conference in 2014. We wish to direct the attention of professional librarians and the academic community in the region towards better sharing and management of information regarding their own institutions’ research activities.

## Conclusion

KAUST also believes that good practices in research data management (RDM) are going to be a key part of responsible research moving forward. We expect to have an increasing role in facilitating the management and sharing of research data in ways aligned with the KAUST mission and beneficial to the global research community. There remain numerous questions to address regarding data storage, sharing, reuse and citation, along with issues of varied institutional and funder requirements in terms of data governance. Services and initiatives around data infrastructure, stewardship, and management support will be important next steps for KAUST in connecting the pieces of scholarly communication.

## Data availability

The data referenced by this article are under copyright with the following copyright statement: Copyright: © 2015 Baessa M et al.

Data associated with the article are available under the terms of the Creative Commons Zero "No rights reserved" data waiver (CC0 1.0 Public domain dedication). 

*F1000Research*: Dataset 1. Unique repository materials citations, 10.5256/f1000research.6502.d50203

*F1000Research*: Dataset 2. Bibliometric benchmarking of KAUST University compared to other similar institutions, 10.5256/f1000research.6502.d50204
