# Supplementary material for: Connecting the pieces: Using ORCIDs to improve research impact and repositories
Source: F1000Res. 2015 Jul 7;4:195. [Version 1] doi: 10.12688/f1000research.6502.1 (PMC4654437; doi:10.12688/f1000research.6502.1)
Supplement: Peer review history

# Peer review: Referee response for version 1

**DOI:** 10.5256/f1000research.6978.r10068

**Published:** 2015-09-01

**Authors:** Antonella De Robbio

**Affiliations:** 1 University Centre for Libraries (CAB), University of Padua, Padua, Italy

I will suggest a slight modification of the title from “ *Connecting the pieces: Using ORCIDs to improve research impact and repositories*” to “ *Connecting the pieces to improve research impact and repositories: Moving towards use of a CRIS system*”

The abstract provides an adequate summary of the article.

The paper is coherent and good structured, comprehensive explanation of argument treated. Level of writing is appropriate for journal’s audience, also regarding the style and terminology, it is clear it gives a good overview on management and organizational aspects. The paper offers a good level on overviews on IR inside Open Access context: principles, strategy, channels and tools are good defined.  Information on policy and infrastructures are very useful for other institutions. Data and figures presented updated and well embedded inside the context. Results are presented accurately and the conclusions are justified and supported by the data.

I have read this submission. I believe that I have an appropriate level of expertise to confirm that it is of an acceptable scientific standard.

# Peer review: Referee response for version 1

**DOI:** 10.5256/f1000research.6978.r9385

**Published:** 2015-07-13

**Authors:** Sarah L. Shreeves

**Affiliations:** 1 University of Miami, Coral Gables, FL, USA

This article outlines the infrastructure in place at KAUST to support both research outputs, particularly those that are not formally published, and research metrics. While the tools themselves are not new or unique, the implementation at a very young university (established in 2009) and the integration of ORCID into multiple systems makes this article acceptable for the Software Tool section.

The abstract is appropriate. The datasets for figures were provided.The article itself is descriptive rather than research, but provides the appropriate context and information for understanding how the systems and tools interact. 

I would suggest a small change to the title which concentrates on ORCID - the article itself is a bit more broad ranging than that and it might be more appropriate to have a broader title.

I have read this submission. I believe that I have an appropriate level of expertise to confirm that it is of an acceptable scientific standard.
